# Tumor-infiltrating immune cells and survival in head and neck squamous cell carcinoma: a retrospective computational study

**DOI:** 10.1038/s41598-024-56738-3

**Published:** 2024-03-16

**Authors:** Lei Zhang, Wei-Quan Wang, Jun-Hong Chen, Jia Feng, Ya-Zhou Liao, You Zou, Rong Liu

**Affiliations:** 1 Department of Clinical Pharmacology, Xiangya Hospital, Central South University, 87 Xiangya Road, Changsha, 410008 People’s Republic of China; 2 Institute of Clinical Pharmacology, Hunan Key Laboratory of Pharmacogenetics, Central South University, 110 Xiangya Road, Changsha, 410078 People’s Republic of China; 3 Engineering Research Center of Applied Technology of Pharmacogenomics, Ministry of Education, 110 Xiangya Road, Changsha, 410078 People’s Republic of China; 4 National Clinical Research Center for Geriatric Disorders, 87 Xiangya Road, Changsha, 410008 Hunan People’s Republic of China; 5 Department of Oral and Maxillofacial Surgery, Center of Stomatology, Xiangya Hospital, Central South University, 87 Xiangya Road, Changsha, 410008 People’s Republic of China; 6 High Performance Computing Center, Central South University, Changsha, 410008 People’s Republic of China

**Keywords:** Tumor-immune infiltration, HNSCC, Tumorigenic site, Survival, Immune-related genes, Cancer, Computational biology and bioinformatics, Immunology, Biomarkers, Medical research, Oncology

## Abstract

The immune infiltration profiles of the tumor microenvironment have effects on the prognosis of head and neck squamous cell carcinoma (HNSCC). Whereas, HNSCC is a heterogeneous group of tumors, but past work has not taken this into consideration. Herein, we investigate the associations between survival and the function of immune cells in different tumorigenic sites of HNSCC. 1149 samples of HNSCC were collected from publicly accessible databases. Based on gene expression data, CIBERSORTx was applied to determine the proportion of 22 immune cell subpopulations. In the Cox regression model, the associations between overall survival, disease-free survival, and immune cells were examined, modeling gene expression and immune cell proportion as quartiles. Consensus cluster analysis was utilized to uncover immune infiltration profiles. Regardless of tumor sites, CD8+ T cells and activated CD4 memory T cells were associated with favorable survival, while eosinophils were the opposite. The survival of the hypopharynx, oral cavity, and larynx subsites was somewhat affected by immune cells, while the survival of the oropharynx subsite potentially was the most impacted. High expression of TIGIT, CIITA, and CXCR6 was linked to better survival, mainly in the oropharynx subsite. Immune cell clusters with four distinct survival profiles were discovered, of which the cluster with a high CD8+ T cell content had a better prognosis. The immune-infiltration pattern is related to the survival of HNSCC to varying degrees depending on the tumor sites; forthcoming studies into immune-mediated infiltration profiles will lay the groundwork for treating HNSCC with precision therapy.

## Introduction

The most prevalent kind of head and neck cancer to affect the upper respiratory system and digestive tract is head and neck squamous cell carcinoma (HNSCC)^1^, whose occurrence is influenced by human papillomavirus (HPV) infection, chronic alcohol use, and cigarette use^2^. In the past few years, there has been a decline in the occurrence of HNSCC attributed to the decrease in smoking rates in many affluent nations. Nevertheless, the prevalence of oropharyngeal squamous cell carcinoma has risen concurrently, with HPV infection being a significant contributing factor to this trend^3^. Interestingly, patients with HPV-positive oropharyngeal squamous cell carcinoma had a better prognosis^4^, which may be associated with increased tumor immunity.

Currently, surgery, chemotherapy, and radiotherapy have good curative effects on patients with early-stage HNSCC, but more than 65% of patients with HNSCC still have recurrence and distant metastases^5^. Immunotherapy, particularly PD-1 immune checkpoint inhibitors (ICIs), has greatly improved outcomes in patients with relapse or distant metastases^6^. Pembrolizumab and nivolumab both demonstrated enhanced 6-month progression-free survival (PFS) and overall survival (OS), along with a decreased frequency of serious adverse events for patients with metastatic and recurrent HNSCC^7,8^. Thus, immunotherapy offers a lot of potential for the treatment of HNSCC. Less than 20% of patients, nevertheless, have a long-lasting benefit from targeted immunotherapy^9^. Given the location and histological specificity of HNSCC’s treatment, as well as the complexity of the tumor immune microenvironment, it is vital to completely comprehend the immune infiltration profile of HNSCC to enhance the therapeutic efficacy of ICIs and improve the overall treatment of the disease.

The larynx, pharynx, and oral cavity are the primary sites of genesis for HNSCC^10^. The prevalence and prognosis of HNSCC are location- and tissue-specific^5^. In Europe, the overall prevalence of laryngeal, oral cavity, oropharyngeal, and hypopharyngeal squamous cell carcinomas decreased in sequence, as did the 5-year relative survival^11^. According to the evidence, distinct immune infiltration profiles may have occurred in distinct sites of HNSCC. T-cell infiltration score and regulatory T cells (Tregs) infiltration were found to differ in laryngeal, oral, oropharyngeal, and hypopharyngeal squamous cell carcinomas by Mandal et al.^12^. The interaction between B cells and CD8+ T cells will affect the development of oropharyngeal squamous cell carcinoma^13^. Given these, there is a current gap in the literature regarding the association between tumor immune-infiltration profile and prognosis based on tumor site, which will act as a novel prescription for the treatment of HNSCC.

Herein, comprehensive research has been done at several tumorigenic locations on the immunological landscape of HNSCC, 22 immune cell subtypes, immune-related genes, and their relationships with OS, disease-free survival (DFS), and chemotherapeutic treatment responsiveness. Based on combined cellular gene expression information from 1,149 HNSCC patients from 7 datasets, a computational framework called CIBERSORTx^14^ was applied to accurately figure out the immune cell proportion from the gene expression profiles. Utilizing the consensus cluster, four distinct immune-infiltration modes of HNSCC were investigated. It will be useful for the research of the immunological mechanisms of HNSCC to have a thorough understanding of the function of immune cells in various tumorigenic sites.

## Results

### Clinical features and prognostic differences in tumor subsites

Detailed clinical information was summarized in Table S2a. A total of 899 patients in this research had unambiguous age information; of these, 747 (83.1%) were older than or equal to 50 years old and 152 (16.9%) were under 50. In addition, 214 women (24.1%) and 673 men (75.9%) were included in this study, which showed a certain gender difference. As for histologic grade, there were 507 cases (76.0%) of G1 or G2, 160 cases (24.0%) of G3 or G4. However, gender and grade were not statistically significant in the univariate Cox regression model, and age was significant only in the univariate Cox regression model with OS as the clinical endpoint (Table S4a).

In terms of risk factor statistics, there were 114 HPV-positive patients and 377 HPV-negative patients. Survival analysis for history of HPV infection suggested significant differences in OS (*p* = 1.10 × 10^−2^, Supplementary Fig. S1a) and DFS (*p* = 2.20 × 10^−3^, Supplementary Fig. S1b), not distinguishing tumor subsites. The univariate Cox regression model with OS and DFS as outcomes exhibited no discernible difference in survival between 63 patients without a smoking history and 636 patients with a smoking history. There were 586 patients who had previously consumed alcohol and 193 who had never done so. Differences in DFS were revealed by a survival analysis for drinking history (*p* = 9.60 × 10^−3^, Supplementary Fig. S1c), but there were no differences in OS (Table S4a).

Finally, the tumor subsites were the hypopharynx, larynx, oral cavity, and oropharynx in this study, whose survival analysis showed a statistical difference in DFS (*p* < 1 × 10^−4^, Supplementary Fig. S1d) and statistical difference in OS (*p* = 1.30 × 10^−2^, Supplementary Fig. S1e). Especially the larynx subsite had longer DFS.

### Performance of CIBERSORTx in estimating tumor-immune infiltration

**Figure 1:**
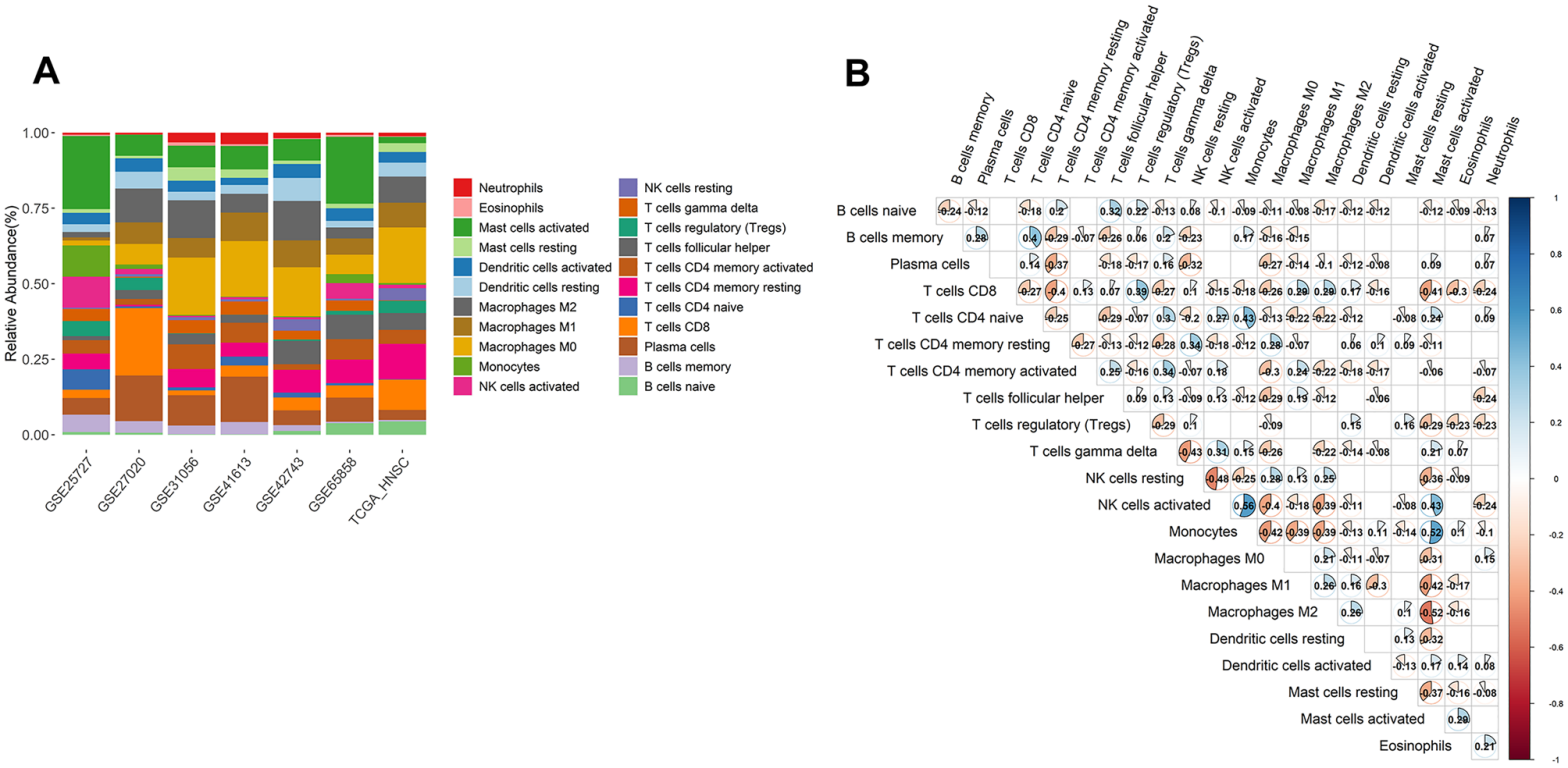
Summary of inferred 22 tumor-infiltrating immune cell subset strata by study. (**A**) Bar chart of the relative content of 22 immune cells in seven datasets; (**B**) Heatmap of correlations between 22 immune cells. Correlation coefficients of *p* < 0.05 are shown.

In this study, the average proportion of genes in the 7 datasets included in the LM22 gene file downloaded from CIBERSORTx was 92.1% (Supplementary Fig. S2). The proportion of samples with a relative abundance of 0 for all immune cell subtypes was less than 80%, so all immune cell subtypes were retained for subsequent analysis (Supplementary Fig. S3). The infiltration of tumor-immune cells in the various datasets was displayed in a bar graph (Fig. 1A). Moreover, M0 macrophages (mean = 12.40%, standard deviation (SD) = 6.67%) and eosinophils (mean = 0.48%, SD = 0.32%) had the highest and lowest relative proportions of immune cell subsets in the tumor immune-infiltration data, respectively.

The 22 immune cell subsets revealed modest to moderate associations when analyzed in paired fashion (Fig. 1B). The most potent and significant relationship (Pearson correlation coefficient (r) = 0.56, *p* = 4.11 × 10^−95^, Table S3) was discovered between monocytes and activated natural killer cells, whereas the sharpest opposite relationship (*r* = − 0.52, *p* = 6.56 × 10^−81^, Table S3) was found between M2 macrophages and activated mast cells.

### Prognosis is impacted by immune cell subsets

**Figure 2:**
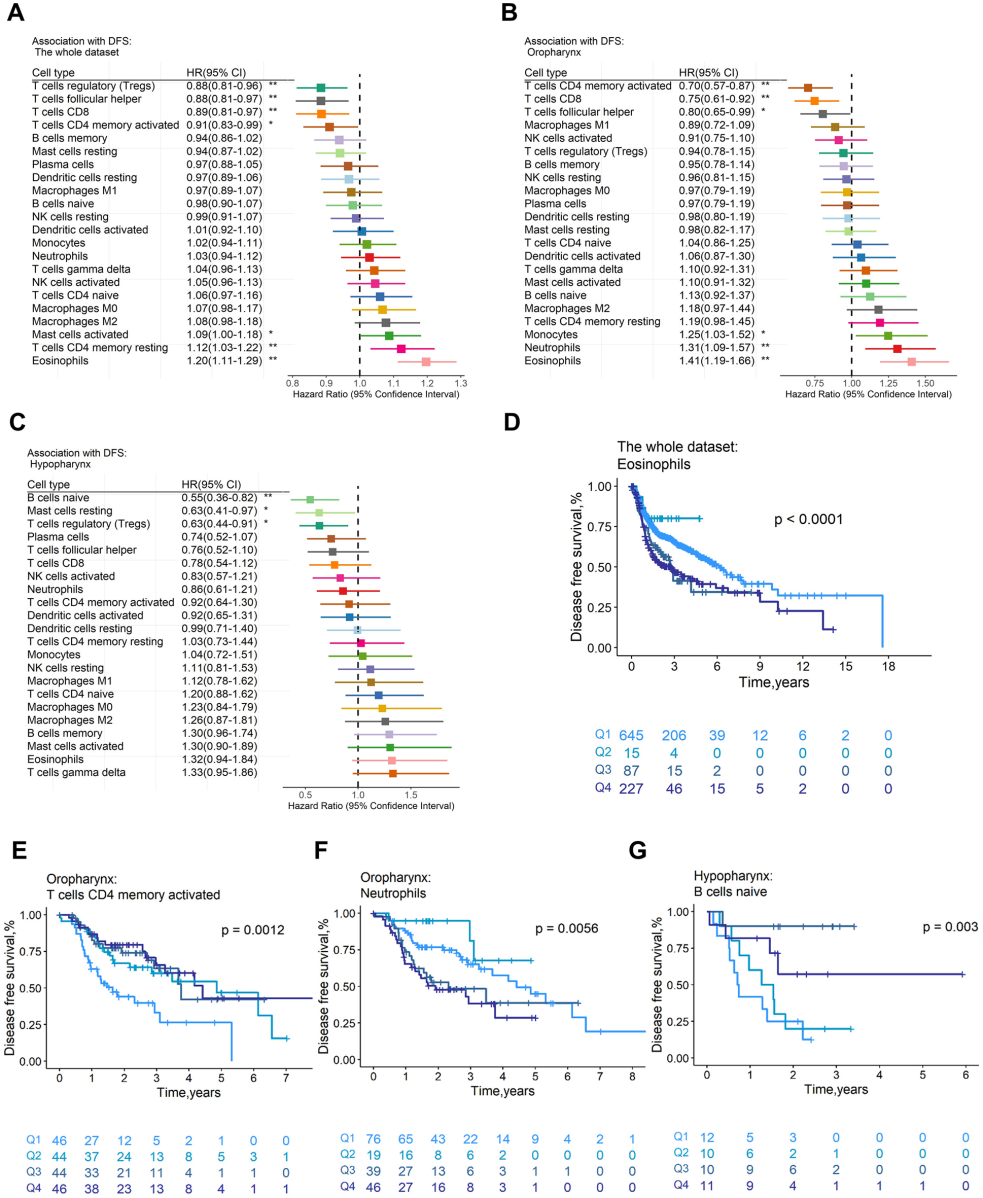
Association of immune cells with disease-free survival across the whole dataset and tumor subsites. Unadjusted HRs (boxes) and corresponding 95% confidence intervals (horizontal lines) for immune cell subtypes about the whole dataset (**A**), oropharynx subsite (**B**), and hypopharynx subsite (**C**). In the survival curves, immune cell subtypes are stratified as quartiles (**D**–**G**). *P*-values are determined by the log-rank tests. HR, hazard ratio. **, *p* < 0.01; *, *p* < 0.05.

**Figure 3:**
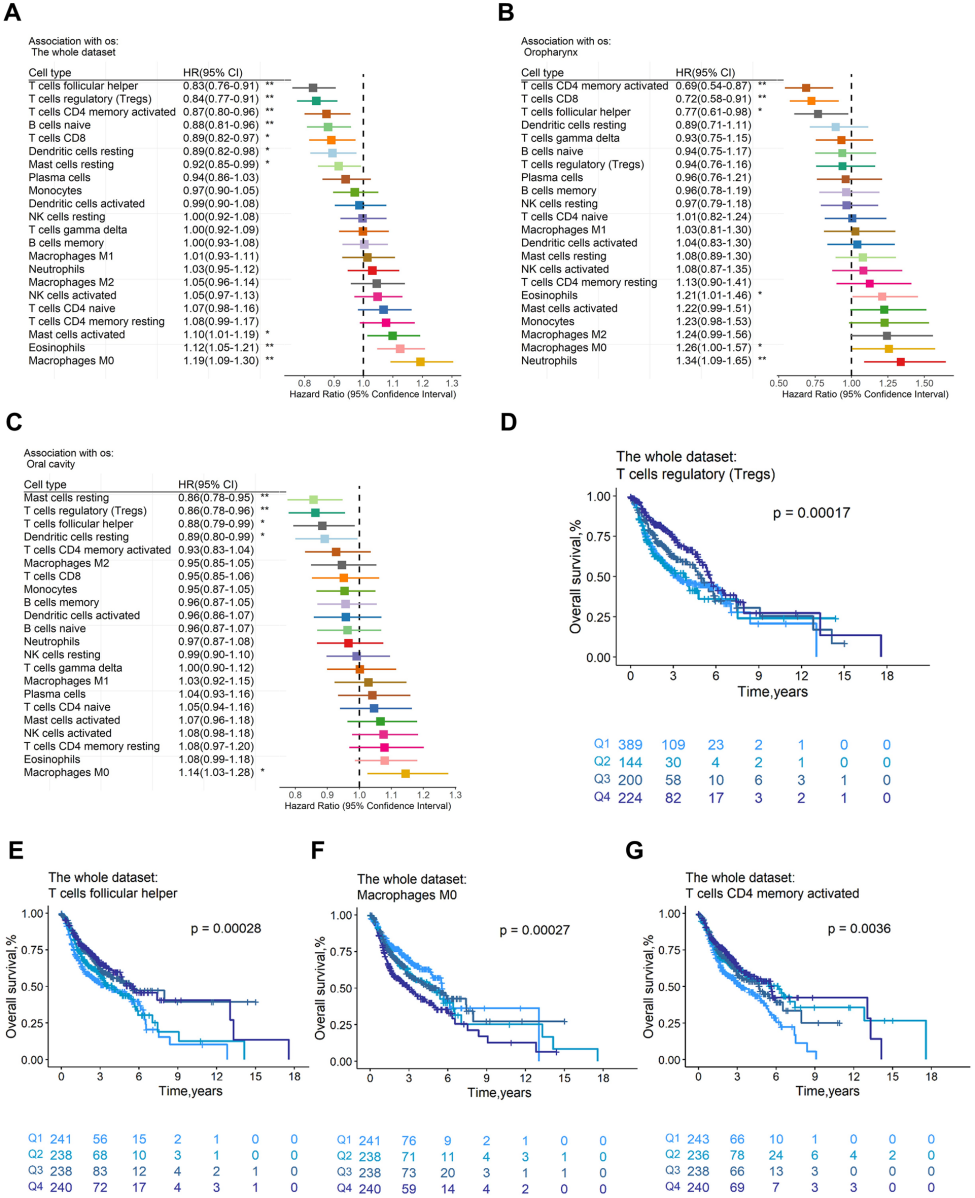
Association of immune cells with overall survival across the whole dataset and tumor subsites. Unadjusted HRs (boxes) and corresponding 95% confidence intervals (horizontal lines) for immune cell subtypes about the whole dataset (**A**), oropharynx subsite (**B**), and oral cavity subsite (**C**). In the survival curves, immune cell subtypes are stratified as quartiles (**D**–**G**). *P*-values are determined by the log-rank tests. HR, hazard ratio. **, *p* < 0.01; *, *p* < 0.05.

The prognosis of individuals with HNSCC is correlated with the percentage of tumor-immune infiltrating cell subgroups. 957 samples with OS (median OS: 2.08 years) and 974 samples with DFS (median DFS: 1.83 years) remained after samples with a CIBERSORTx p value of higher than 0.05, missing survival events, or a survival time of 0 were eliminated. For DFS (Fig. 2A) and OS (Fig. 3A), the hazard ratios (HRs) and 95% confidence intervals (CIs) for immune cell subsets were presented in forest plots. Specifically, the presence of Tregs (DFS: HR = 0.88, *p* = 4.68 × 10^−3^, Supplementary Fig. S4c; OS: HR = 0.84, *p* = 2.86 × 10^−5^, Fig. 3D), CD8+ T cells (DFS: HR = 0.89, *p* = 7.43 × 10^−3^, Supplementary Fig. S4d; OS: HR = 0.89, *p* = 1.06 × 10^−2^, Supplementary Fig. S5c), and follicular helper T (Tfh) cells (DFS: HR = 0.88,* p* = 6.73 × 10^−3^, Supplementary Fig. S4e; OS: HR = 0.83,* p* = 3.28 × 10^−5^, Fig. 3E) were all positively correlated with DFS and OS. However, eosinophils were associated with negative DFS (HR = 1.20,* p* = 1.40 × 10^−6^, Fig. 2D) and OS (HR = 1.12, *p* = 1.39 × 10^−3^, Supplementary Fig. S5d). In addition, we found M0 macrophages were linked to poor OS (HR = 1.19,* p* = 1.01 × 10^−4^, Fig. 3F), whereas naive B cells (HR = 0.88, *p* = 3.21 × 10^−3^, Table S5f) and resting dendritic cells (HR = 0.89, *p* = 1.28 × 10^−2^, Table S5f) were linked to favorable OS. Activated CD4 memory T cells were connected with good DFS (HR = 0.91, *p* = 3.83 × 10^−2^, Table S5a) and OS (HR = 0.87, *p* = 3.15 × 10^−3^, Fig. 3G), while activated mast cells were associated with poor DFS (HR = 1.09, *p* = 4.93 × 10^−2^, Table S5a) and OS (HR = 1.10,* p* = 2.34 × 10^−22^, Table S5f). The survival rates of almost all the immune cells mentioned above were statistically significant between the relative content quartiles. Furthermore, the trustworthiness of our analysis was demonstrated by the HRs and related 95% CIs of the aforementioned significant immune cell subgroups for DFS and OS in distinct datasets, which were displayed in Fig. S9 and S10, respectively.

Moreover, eosinophils remained implicated in DFS by multivariate Cox regression analysis with clinical covariates adjusted (tumor subsites, history of HPV infection, and alcohol consumption) (HR = 1.22, *p* = 2.18 × 10^−3^, Table S6a). Eosinophils also showed an impact on OS in the multivariate Cox regression model on OS after adjusting age and history of HPV infection, (HR = 1.28, *p* = 2.42 × 10^−3^, Table S6g). The HR values of eosinophils were the largest among all immune cell subsets in multivariate Cox regression models based on penalized maximum likelihood estimation with DFS (HR = 1.17, Table S6f) and OS (HR = 1.23, Table S6l) as clinical endpoints, respectively. Similarly, the HR values of activated CD4 memory T cells were lowest (DFS: HR = 0.91, Table S6f; OS: HR = 0.87, Table S6l). Nevertheless, no association was found between immune content score and OS or DFS.

### The profile of immune infiltration is different among tumor subsites

We investigated how immune cells affected the prognosis of four different tumor subsites, including the hypopharynx, larynx, oral cavity, and oropharynx, and discovered that the oropharynx subsite may be most impacted by immune cells. Meanwhile, the four subsites differ in both directions regarding the impact of immune cells on the prognosis for OS and DFS.

For DFS (Fig. 2B) and OS (Fig. 3B), forest plots showed the HRs and 95% CIs for the oropharynx subsite. Activated CD4 memory T cells (DFS: HR = 0.70, *p* = 1.34 × 10^−3^, Fig. 2E; OS: HR = 0.69, *p* = 2.12 × 10^−3^, Supplementary Fig. S5e), CD8 + T cells (DFS: HR = 0.75, *p* = 4.68 × 10^−3^, Supplementary Fig. S4g; OS: HR = 0.72, *p* = 6.05 × 10^−3^, Supplementary Fig. S5f) and Tfh cells (DFS: HR = 0.80, *p* = 4.16 × 10^−2^, Supplementary Fig. S4h; OS: HR = 0.77, *p* = 3.15 × 10^−2^, Table S5g) were generally related to better OS and DFS, whereas neutrophils (DFS: HR = 1.31, *p* = 3.45 × 10^−3^, Fig. 2F; OS: HR = 1.34, *p* = 6.09 × 10^−3^, Supplementary Fig. S5g) and eosinophils (DFS: HR = 1.41,* p* = 5.13 × 10^−5^, Supplementary Fig. S4i; OS: HR = 1.21, *p* = 4.24 × 10^−2^, Table S5g) were linked to worse DFS and OS. In addition, the cells with negative DFS were monocytes (HR = 1.25, *p* = 2.55 × 10^−2^, Table S5b), and the cells with negative OS were M0 macrophages (HR = 1.26, *p* = 4.69 × 10^−2^, Table S5g). After adjusting for clinical variables (age, history of HPV infection and alcohol consumption), the results of the multivariate Cox regression model showed that activated CD4 memory T cells still had favorable effects on DFS (HR = 0.70, *p* = 2.05 × 10^−2^, Table S6b). Moreover, the HR value of activated CD4 memory T cells (HR = 0.72, Table S6f) was the lowest in the multivariate Cox regression model with penalised maximum likelihood estimation. In multivariate Cox regression models for OS adjusted for age, no immune cells had a statistically significant association with OS (Table S6h). When using multivariable Cox regression models with penalized maximum likelihood estimation, the HR value of activated CD4 memory T cells (HR = 0.85, Table S6l) was the lowest.

In the hypopharynx subsite (Fig. 2C), Tregs (HR = 0.63, *p* = 1.23 × 10^−2^, Supplementary Fig. S4j) and resting mast cells (HR = 0.63, *p* = 3.61 × 10^−2^, Table S5c) were associated with good DFS. Naive B cells were related to better DFS (HR = 0.55, *p* = 3.59 × 10^−3^, Fig. 2G) and OS (HR = 0.56, *p* = 8.18 × 10^−3^, Supplementary Fig. S5h). Naive B cells were still favorable to DFS (HR = 0.62, *p* = 3.08 × 10^−2^, Table S6c) and OS (HR = 0.59, *p* = 2.07 × 10^−2^, Table S6j) in the multivariate Cox regression model.

In the oral cavity subsite (Fig. 3C), Tregs (HR = 0.86, *p* = 4.54 × 10^−3^, Supplementary Fig. S5i), Tfh cells (HR = 0.88, *p* = 2.59 × 10^−2^, Table S5i), and resting dendritic cells (HR = 0.89, *p* = 3.96 × 10^−2^, Table S5i) were related to favorable OS, while M0 macrophages (HR = 1.14, *p* = 1.63 × 10^−2^, Supplementary Fig. S5j) were related to adverse OS. Resting mast cells were associated with good DFS (HR = 0.88, *p* = 3.78 × 10^−2^, Table S5d) and OS (HR = 0.86,* p* = 2.56 × 10^−3^, Supplementary Fig. S5k). Statistically, no immune cells were associated with DFS (Table S6e) in the multivariate Cox regression analysis model, but Tregs were linked to OS (HR = 0.80, *p* = 1.06 × 10^−2^, Table S6i).

The results of the larynx subsite were presented in the supplementary Fig. S4b and Fig. S5b. Tfh cells were associated with better DFS (HR = 0.84, *p* = 3.61 × 10^−2^, Table S5e) and OS (HR = 0.73,* p* = 7.10 × 10^−3^, Table S5j) in the larynx subsite in this study. Tfh cells (HR = 0.85, Table S6l) contributed to better OS in the multivariate Cox regression model with penalised maximum likelihood estimation. Moreover, no association was found between immune content scores and OS or DFS across tumor subsites (Table S9).

### Immune clusters linked to tumor subsites and clinical outcomes

**Figure 4:**
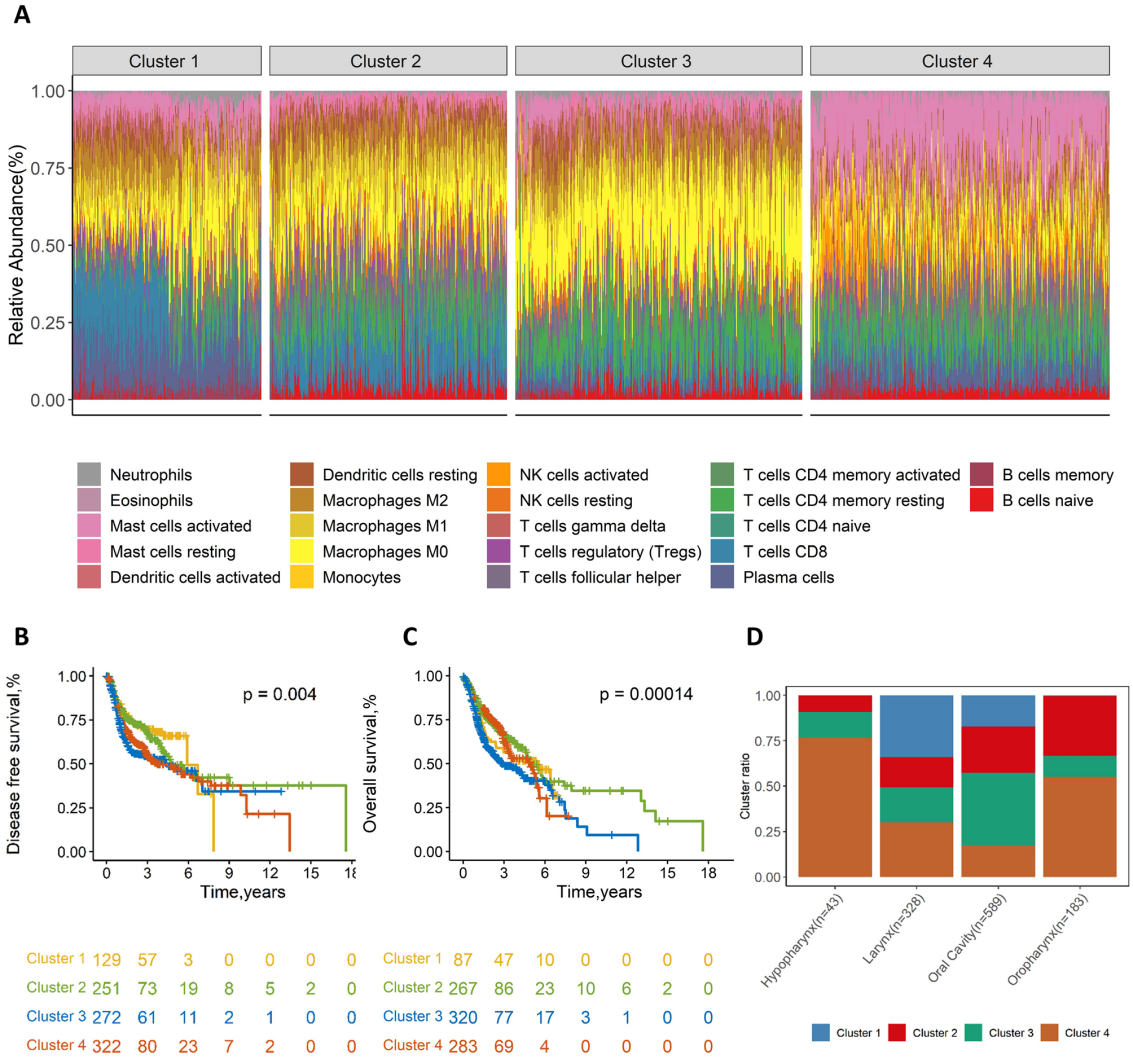
Consensus clustering of all samples based on immune cell relative abundance and survival curves by clusters. Stacked bar charts of samples ordered by cluster assignment (**A**). Survival curves for DFS (**B**) and OS (**C**) by cluster. Bar graphs showed the proportion of immune clusters in the four tumor subsites (**D**). *P*-values are determined by the log-rank tests. DFS, disease-free survival; OS, overall survival.

Consensus clustering was used to see if there were distinct immunologic tumor infiltrating profiles, so we identified four distinct immune clusters based on the kinds of HNSCC immunological tumor infiltrating cells. Each immune cell subset in the four immune clusters was compared in Fig. S6, and Fig. 4A showed the percentage of tumor-immune infiltrating cells in each of the immunological clusters**.** The survival rates with respect to DFS (*p* = 4.00 × 10^−3^, Fig. 4B) and OS (*p* = 1.40 × 10^−4^, Fig. 4C) were significantly different between immune clusters by log-rank test, and pairwise comparisons between clusters were exhibited in Fig. S7. Cluster 1 was predominantly composed of CD8+ T cells and plasma cells; Cluster 2 was characterized by a greater percentage of CD8+ T cells, activated CD4 memory T cells, Tregs, and Tfh cells; Cluster 3 showcased a high ratio of M0 macrophages and resting CD4 memory T cells; In cluster 4, monocytes and activated mast cells played a significant role. We found a significant difference in DFS (*p* = 1.10 × 10^−2^, Supplementary Fig. S7d) and OS (*p* < 1.0 × 10^−4^, Supplementary Fig. S7g) between immune cluster 2 and immune cluster 3, apparently as a result of cluster 2 having a larger percentage of immune cells linked to favorable DFS and OS, such as activated CD4 memory T cells, Tregs, and Tfh cells (Supplementary Fig. S7).

Furthermore, we examined the distribution of immune clusters among subsites and were surprised to find that cluster 1 were absent in the hypopharynx subsite (Fig. 4D). Cluster 2 wasthe main subtypes of the hypopharynx. Cluster 1 and Cluster 4 were the most common subtypes of larynx. Cluster 3 was predominant in the oral cavity. Cluster 2 and cluster 4 were the most common in the oropharynx subsite.

Overall, the above results revealed that different profiles of tumor immune infiltration existed in different subsites of HNSCC, resulting in differences in prognosis.

### Immune-related genes are prognostic

**Figure 5:**
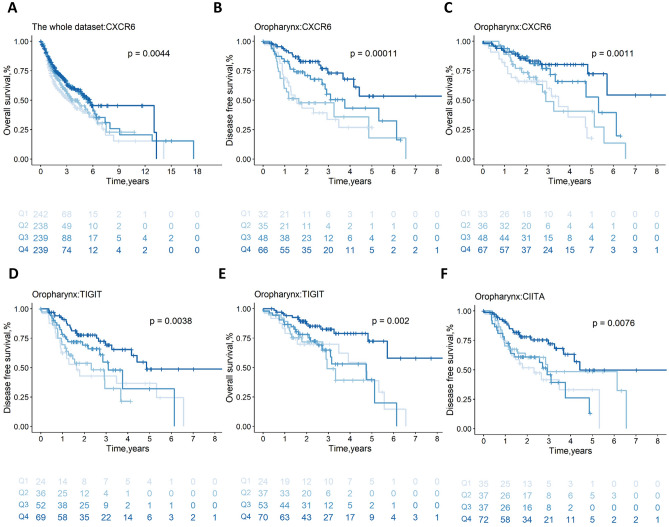
The association of immune gene expression with prognosis is illustrated by survival curves. Survival curve plots for CXCR6 gene expression versus OS (**A**) in the whole dataset; survival curve plots for CXCR6 (**B**, **C**), and TIGIT (**D**, **E**) gene expression versus DFS and OS in the oropharynx subsite, respectively; survival curve plots for CIITA (**F**) gene expression versus DFS in the oropharynx subsite. *P*-values are determined by the log-rank tests. DFS, disease-free survival; OS, overall survival.

It was investigated if immune-related genes linked to immune cells or immunological checkpoints were correlated with the OS and DFS of HNSCC patients. Firstly, we found the strongest positive correlation between TIGIT and CXCR6 gene expression (*r* = 0.81, *p* = 2.35 × 10^−245^, Table S7c) and the weakest positive correlation between CMTM6 and CXCR6 gene expression (*r* = 0.28, *p* = 7.51 × 10^−22^, Table S7c). Next, without discriminating between subsites, high expression of CIITA (DFS: HR = 0.88, *p* = 7.81 × 10^−3^, Supplementary Fig. S8a; OS: HR = 0.88,* p* = 5.53 × 10^−3^, Supplementary Fig. S8b), CXCR6 (DFS: HR = 0.88, *p* = 3.80 × 10^−3^, Supplementary Fig. S8c; OS: HR = 0.85, *p* = 4.30 × 10^−4^, Fig. 5A) and TIGIT (DFS: HR = 0.88, *p* = 1.01 × 10^−2^, Supplementary Fig. S8e; OS: HR = 0.89, *p* = 7.85 × 10^−3^, Supplementary Fig. S8f) was associated with favorable OS and DFS, while high expression of CMTM6 was associated with unfavorable DFS (HR = 1.11, *p* = 2.73 × 10^−2^, Supplementary Fig. S8g). The expression of STAT3 was not statistically associated with DFS (Table S7a) or OS (Table S7b).

In the oropharynx subsite, we discovered high expression of CXCR6 (DFS: HR = 0.68, *p* = 1.20 × 10^−4^, Fig. 5B; OS: HR = 0.64,* p* = 3.04 × 10^−4^, Fig. 5C) and TIGIT (DFS: HR = 0.74, *p* = 3.29 × 10^−3^, Fig. 5D; OS: HR = 0.71,* p* = 3.34 × 10^−3^, Fig. 5E) was linked to better OS and DFS. Additionally, high levels of CIITA (HR = 0.79,* p* = 1.68 × 10^−2^, Fig. 5F) were associated with positive DFS in the oropharynx subsite. In the other subsites, no statistical correlation was found between DFS (Table S7a) and OS (Table S7b) and the above immune-related genes, respectively.

### The prognostic role of immune cell responses to chemotherapeutic agents

Using drug response information from TCGA, the correlation between immune cells and drug response was explored (Table S10). Overall, there is no statistically significant correlation between immune cell subtypes and complete responses, which might be explained by a limited sample size, but these correlations could be used to interpret the correlation between monocytes (odds ratio (OR) = 0.67, *p* = 9.10 × 10^−2^) or resting dendritic cells (OR = 0.61, *p* = 8.10 × 10^−2^) and beneficial drug responses. Due to this, a multivariate analysis was omitted.

## Discussion

In this work, we collected bulk gene expression data from 1149 individuals with HNSCC and made use of CIBERSORTx to estimate the relative proportions of 22 immune cell subsets. Following that, we looked at how immune clusters, immune cell proportions, and immune-related gene expression correlated with the survival of HNSCC in various tumor subsites. Increased activated CD4 memory T cells and CD8+ T cells contribute to favorable clinical outcomes, whereas eosinophils do the opposite. Importantly, the profile of tumor immune infiltration also varies significantly according to its subsites, with the oropharynx subsite potentially the most impacted.

In general, CD4+ T cells not only have direct cytotoxic effects but also affect angiogenesis and the secretion of cytokines^15^. They are closely associated with other lymphocytes and non-lymphocyte cells, including recruiting neutrophils, enhancing B cell maturation, and boosting phagocytosis in macrophages^15^. Meanwhile, CD4+ T cells with cytolytic ability showed significant antitumor activity, mainly through recognition of MHC-II and releasing INF-γ^16^. Tfh cells, one of the significant subsets of CD4+ T cells, contributed to favorable OS and DFS in the study. Tfh cells induce B cells to initiate antibody responses outside follicles and germinal centers, express the chemokine CXCL13 to recruit CD8 + T cells for anti-tumor immunity, and dedicate themselves to the efficacy of anti-PD-L1 and PD-1^17,18^. Yet, the specific anti-tumor mechanism is not very clear in HNSCC, which is worth our further study. We noticed that CD8+ T cells and B cells contributed to better outcomes in patients with HNSCC in this research. This suggests that it may be valuable to further explore the crosstalk between Tfh cells and B cells, CD8 + T cells, and even other immune cells in HNSCC. We found that different status of CD4+ T cells have different effects. In this study, naive CD4 + T cells and resting CD4 memory T cells are associated with poor prognosis, while activated CD4 memory T cells are on the contrary. The same was discovered by Yu et al^19^. We can't ignore the status of immune cells when studying their role. In a preclinical HNSCC model, Shibata et al^20^. increased the effectiveness of a tumor vaccination by integrating CD4+ T cells, which may suggest that appropriate vaccines or drugs can be designed to increase the proportion of activated CD4 memory T cells to effectively exert antitumor effects.

Eosinophils have tumor-promoting and antitumor effects, depending on differences in the cytokines or chemokines^21^. Eosinophils express IL-10, which impedes the development of prostate cancer cells in culture^22^, but eosinophils can be activated by tumor-derived thymic stromal lymphopoietin (TSLP), thereby increasing the expression of IL-4, IL-5, and IL-13 and stimulating the growth of cervical cancer cells^23^. Of note, we observed that eosinophils were connected to adverse OS and DFS in the whole dataset and oropharynx subsite. More investigations have revealed that eosinophils are linked to a worse prognosis, even if their function in HNSCC is somewhat controversial. Lee et al. found that eosinophils promote angiogenesis and tumor migration via the chemokine CCL2^24^. More investigation is crucial to completely comprehend the role of eosinophils in HNSCC.

Given that every coin has two sides, Tregs in our study were found to be inconsistent with their accepted roles. Tregs often act as accomplices in tumor immune escape and often adversely affect clinical outcomes^16^. By contrast, we found that Tregs were associated with DFS, especially in the hypopharynx subsite, but also contributed to favorable OS in the oral cavity subsite. In fact, the relationship between high Tregs recruitment and the prognosis of HNSCC remains controversial^12,25^. One of the reasons for Tregs being a good prognostic marker is that, depending on whether the tissue releases IL-12 and TGF-β, Tregs transform into two distinct immune subpopulations and thus have immunosuppressive or immune-boosting effects^26^. Similarly, neuropilin-1^–/–^ Tregs produce IFN-γ, which drives the fragility of surrounding wild-type Tregs, boosts antitumor immunity, and facilitates tumor clearance^27^. Therefore, it is crucial to continue researching the subpopulation of Tregs or their molecular mechanisms in HNSCC.

CD8+ T cells, also known as cytotoxic T cells, induce antitumor immunity by releasing INF-γ, and are primarily involved in two subgroups in HNSCC, Tc17 and Tc22^28^. We noticed that the high percentage of CD8+ T cells contributed to good OS and DFS, which is consistent with the published literature^29,30^. Hladková et al^13^. uncovered that cell interactions between B cells and CD8+ T cells influence the prognosis of oropharyngeal squamous cell carcinoma. We obtained similar results: in the hypopharynx subsite, naive B cells and plasma cells were associated with good clinical outcomes, possibly due to increased semaphorin 4A expression^31^. Mast cells can promote tumor invasiveness by releasing matrix metalloproteinases and proangiogenic factors^32^. Consistently, resting mast cells were associated with good DFS in the hypopharynx subsite and good OS in the oral cavity subsite, respectively. Although statistically only M0 macrophages were found to be associated with adverse OS in this study, the influence of macrophages on HNSCC cannot be ignored. Tsujikawa et al^33^. observed that macrophages promote HNSCC metastasis to lymph nodes via the CCR4/CCL22 axis in a mouse model.

Our results suggested that high expression of CIITA, CXCR6, and TIGIT was somewhat associated with favorable prognosis, specifically in the oropharynx subsite. Uniformly, Wu et al^34^. found that TIGIT was overexpressed in CD4+ T cells, CD8 + T cells, and Tregs in patients with HNSCC, which is expected to be a new effective immune checkpoint besides PD-1/PD-L1 and CTLA-4. As an important regulatory factor of MHC-II synthesis, CIITA plays an important role in antigen presentation^35^. In lung cancer, or HNSCC, the CXCR6/CXCL16 axis effectively promotes the recruitment of CD8 resident memory T cells and enhances the efficacy of tumor vaccines^36^. CMTM6, as a regulatory factor of the PD-L1 protein, promotes tumor growth through the Wnt/β-catenin signaling pathway, thereby predicting poor prognosis in patients with HNSCC^37^. However, our findings were not statistically significant by log-rank test. Oweida et al^38^. found antagonistic STAT3 to regulate the function of Tregs to enhance radiotherapy efficacy, which was consistent with our results in the oropharynx subsite.

Furthermore, consensus cluster analysis based on immune cell fractions revealed four immune subgroups with different prognoses, with clusters 2 and 3 showing the greatest difference. Since the classification is based on gene expression data, further confirmation by immunohistochemistry is needed. As mentioned above, the molecular mechanisms of different immune subpopulations are explored based on multicellular interactions and multi-omics analysis in the following study.

Focusing on the correlation analysis between immune cell infiltration or immune-related genes and prognosis, there were significant differences in the immune influence of tumors at different sites. The oropharynx subsite may have better immunotherapy benefits, while the larynx subsite may have the opposite effect. There could be some explanations for the disparity between the literature's descriptions of immune infiltration of tumor subsites. Indeed, the prognosis of HPV-positive carcinomas is better than that of HPV-negative tumors, which may be due to enhanced immune infiltration following infection^12^. HPV-positive infection rates are highest in the oropharynx, while the larynx is less susceptible to infection^39^. Moreover, another explanation is that there is richer lymphoid tissue in the oropharynx than in the larynx^12^.

There are some unavoidable limitations to our research. For one thing, we used the deconvolution algorithm to estimate the immune cell proportion rather than the experimental method. Despite minor changes, several investigations have demonstrated the accuracy of the calculating approach, and our results are in line with publications in the literature. For another, our study was retrospective, and some clinical information was missing, such as metastases, lymph nodes, and drug responses, which would reduce the statistical power of the multivariate analysis. More biological experiments or clinical cohorts are required to corroborate our findings.

Collectively, our study revealed profiles of immune infiltration at different sites of HNSCC. Tregs, CD8+ T cells, naive B cells, Tfh cells, activated CD4 memory T cells, and resting mast cells are linked to favorable prognosis, while eosinophils, M0 macrophages, resting CD4 memory T cells, activated mast cells, and naive CD4+ T cells are the opposite. Furthermore, the high expression of CIITA, CXCR6, and TIGIT is prognostically favorable, which could be expected to be new targets. Additionally, we revealed that different subsites in HNSCC have different profiles of immune infiltration, in which the oropharynx subsite may be most affected by immunity but the larynx subsite may be least. In short, our results aim to provide insight into the immune microenvironment at different sites of HNSCC and provide evidence for treatment options at different sites, thus promoting the development of treatment modalities for HNSCC.

## Materials and methods

### Study population

**Figure 6:**
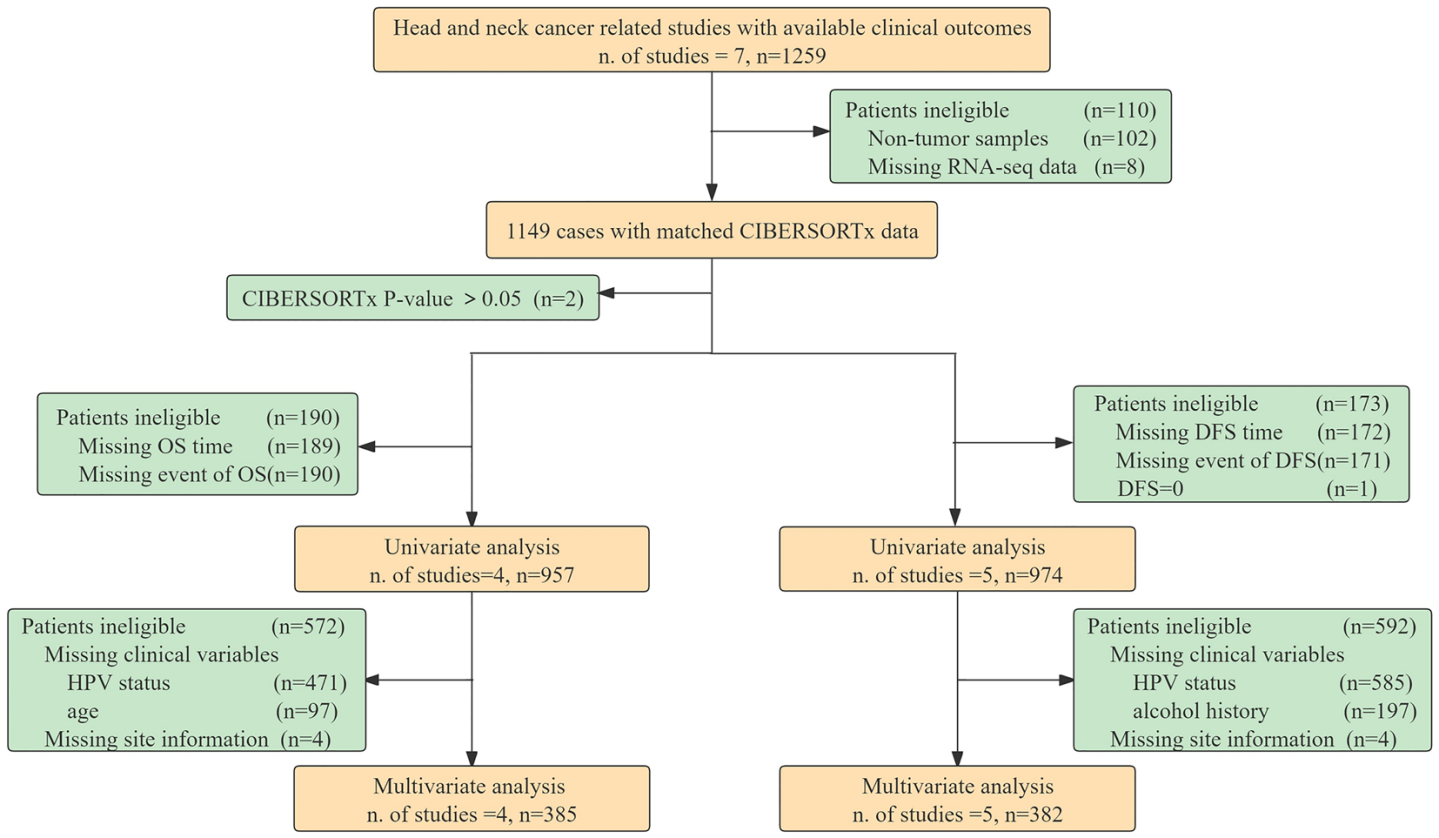
A flowchart of the study that details the samples utilized at each stage of statistical analysis. DFS, Disease free survival; OS, Overall survival.

Publicly accessible databases were consulted to compile information regarding HNSCC. The gene expression data and clinical information were derived from The Cancer Genome Atlas (TCGA, https://portal.gdc.cancer.gov/) and Gene Expression Omnibus (GEO, https://www.ncbi.nlm.nih.gov/geo/) searched with the keywords "head and neck squamous cell carcinoma" or "HNSCC". Datasets without gene expression data or exact clinical details were excluded. In a nutshell, the study includes 1,259 individuals with matching mRNA expression data and clinical outcomes from 7 trials (Table S1a). All research cited in this paper has ethical approval and patient consent statements available in the relevant original publication. Figure 6 displays the sample selection for each stage of the subsequent study and analysis.

### Data processing

The series matrix files (as uploaded by the authors) of the datasets, including GSE27020^40^, GSE31056^41^, GSE41613^42^, GSE42743^42^, GSE25727^43^, and GSE65858^44^, from the GEO database were used to obtain the normalized gene expression data, relevant clinical information, and survival information. Additionally, due to the lack of normalized gene expression data in the series matrix file of GSE42743, the raw gene transcriptome data (.cel) of GSE42743 obtained from GEO were background corrected and normalized by the robust multiarray average (RMA) algorithm^45^, which could effectively remove the variation between samples. Each sample had any probes that couldn't be connected to a particular gene eliminated. When more than one probe is employed to map a gene, the average value of all probes linked to that gene is calculated. Normalized RNA-seq data (TPM values), clinical information, and drug response information for HNSCC from TCGA were downloaded by the R package "TCGAbiolinks"^46^. The survival outcomes summarized by Liu et al^47^. were used in this study. The characteristic distribution of clinical information collected in this study is presented in Table S2a.

### Identification of tumor subsites

According to the anatomical location of the tumor, HNSCC was mainly divided into four subsites, including the hypopharynx, larynx, oral cavity, and oropharynx. The tonsils, base of the tongue, oropharynx, and the like are included in the oropharynx subsite. The buccal mucosa, floor of the mouth, lip, hard palate, oral cavity, and oral tongue are all considered subsites of the oral cavity^48^. The classification for the larynx and hypopharynx is larynx subsite and hypopharynx subsite, respectively^49^. The anatomical sites of all datasets were verified and classified according to this criterion. In Table S2b, the sample sizes for four tumor subsites are displayed, along with the distribution of tumor subsites across various datasets.

### Estimating infiltrating immune cell subsets

In this work, we made full use of CIBERSORTx (https://cibersortx.stanford.edu/)^14^, an analytical device that makes use of gene expression data to infer gene expression profiles and provide an estimate of the relative abundances of various cell types in mixed cell populations.

The relative abundance of 22 immune cells was calculated using gene expression datasets that had been normalized and combined with 547 genes from the LM22 signature matrix. The B-mode, 1000 permutations, and disabling quantile normalization were used to correct for batch effects and obtain statistically robust analyses. The following correlation investigation excluded samples with a deconvolution p value less than 0.05.

### Estimate of immune score and selection of immune genes

We estimated the immune content score using the R package "ESTIMATE"^50^ and identified five immune-related genes (TIGIT^34^, CXCR6^36^, STAT3^38^, CMTM6^37^, and CIITA^35^) based on a literature study to investigate the relationship between the immune content score, immune-related genes, and the prognosis of HNSCC. The scale for the immune content score ranged from 0 to 1.

### Explore the profile of tumor immune cells by consensus clustering

Consensus clustering was performed on immune cell fractions found by CIBERSORTx with samples with a p value of 0.05 or lower with the aim of looking into the profiles of immune cells invading tumors in HNSCC. The relative content of immune cells was scaled between 0 and 1 to improve comparability due to the wide variation in the relative content of the various immune cell subtypes. Applying the "ConsensuClusterPlus" R package^51^ and repeating sampling 1000 times, clustering was carried out to confirm the stability of classification. Meanwhile, Euclidean distance using the k-means algorithm was used. After that, the link between clinical outcomes and clusters of immune cells infiltrating was looked at using univariate Cox hazard analysis and the log-rank test.

## Statistical analyses

The study included clinical information such as age, gender, grade, history of HPV infection, history of smoking, history of alcohol consumption, and drug response. Both DFS and OS were clinical outcomes. DFS is the amount of time that passes between randomization and the onset of a new illness or death. The OS is the amount of time between randomization and death, regardless of the cause. Recurrence-free survival (RFS), progression-free survival (PFS), and progression-free interval (PFI) were used in lieu of DFS in some datasets without DFS. This is the fact that when DFS, RFS, PFS, and PFI are less than OS, they are roughly equivalent under their respective definitions. In addition, drug response information was only available in the HNSC dataset. According to RECIST 1.1 (The Response Evaluation Criteria in Solid Tumors), stable disease (SD) and progressing disease (PD) were classified as non-responders, while the remaining patients were classified as responders. This resulted in binary variables.

If the relative content of immune cell subtypes in 80% of samples was zero, the immune cell subtype would not be included in the subsequent analysis. As continuous variables, the relative content of immune cells was separated into quartiles. To investigate the relationship between immune cells and survival, the Cox regression model was applied. The log-rank test, which was used to assess the interquartile survival rates of the immune cell relative content, was illustrated using Kaplan–Meier survival curves. The Benjamini–Hochberg method adjusted the p values. The study served as the strata variable in the Cox regression models. Similarly, the associations between survival and clinical covariates (age, gender, grade, and so on) were evaluated. The multivariate Cox regression model incorporated clinical variables and immune cell subtypes that were statistically significant in the univariate Cox regression model. Additionally, when confounding is successfully controlled, several parameters from the univariate Cox regression model may result in the removal of strongly connected components. To deal with this, penalized maximum likelihood calculations applying the "glmnet" package in R were used to create multivariable Cox regression models^52^. The penalization factor was established using the results of 1,000 cross-validation experiments.

Moreover, the association between the quartiles of the relative proportion of immune cells and the drug response was determined using logistic regression analysis. To ascertain the connection between category variables, chi-square tests were implemented. The Pearson correlation coefficient was adopted to assess the pairwise correlation between immune cells. The immune-related genes, immunological clusters, and prognosis were all statistically assessed similarly with immune cells.

In this work, R version 4.2.2^53^ was employed for all statistical analyses. All study findings were taken to be statistically significant if the p value was less than 0.05.

### Supplementary Information

Supplementary Figures.

Supplementary Tables.

## Data Availability

The public databases TCGA and GEO contain the datasets needed to support the findings of this work under the accession codes TCGA_HNSC, GSE27020, GSE31056, GSE41613, GSE42743, and GSE25727. The institutional review boards for each of these investigations have already given their prior approval.
