# Supplementary material for: Tumor-infiltrating immune cells and survival in head and neck squamous cell carcinoma: a retrospective computational study
Source: Sci Rep. 2024 Mar 16;14:6390. doi: 10.1038/s41598-024-56738-3 (PMC10944537; doi:10.1038/s41598-024-56738-3)
Supplement: Supplementary file 1 — Supplementary Figures. [file 41598_2024_56738_MOESM1_ESM.docx]

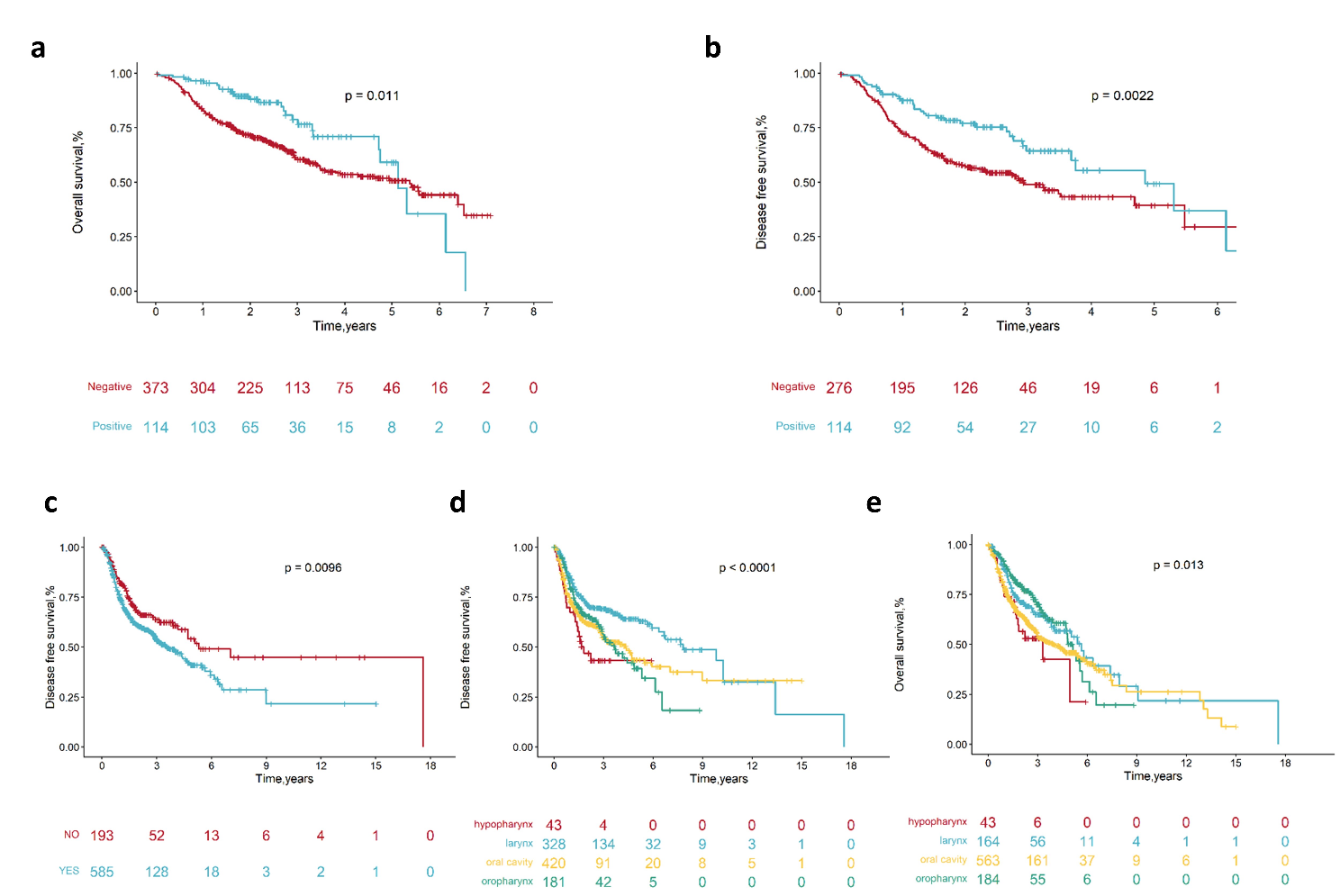
**Figure S1:** Survival curves for different tumor sites and clinical covariates. Survival curve plot for HPV infection and DFS (a) and OS (b); Survival curve plot for alcohol history and DFS (c); Survival curve plots for DFS (c) and OS (d) within different tumor subsites. P-values are determined by the log-rank tests in Kaplan-Meier survival curves. DFS, disease-free survival; OS, overall survival.


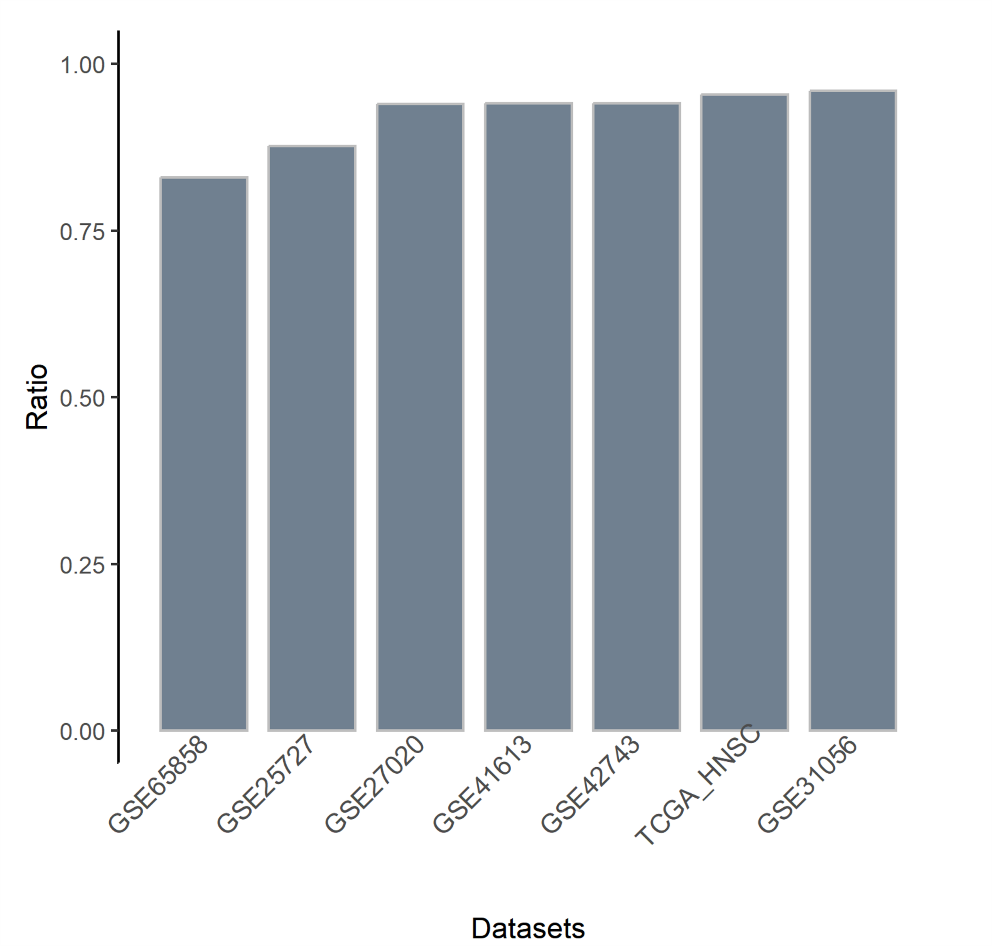


**Figure S2:** The bar chart shows the proportion of the LM22-547 signature matrix genes available by study.


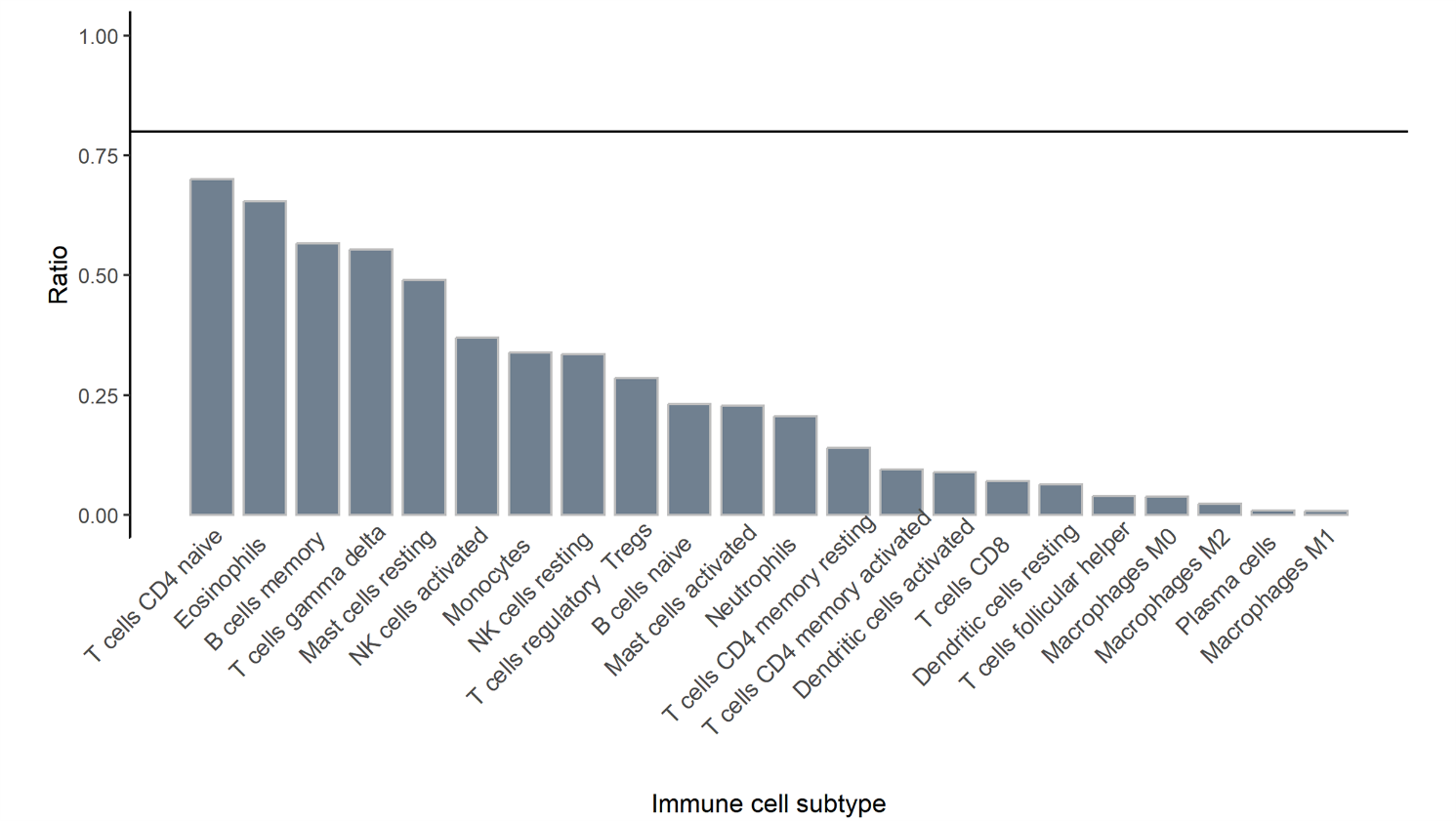


**Figure S3:** The bar chart shows the proportion of samples in which the relative abundance of each immune cell subtype is zero.


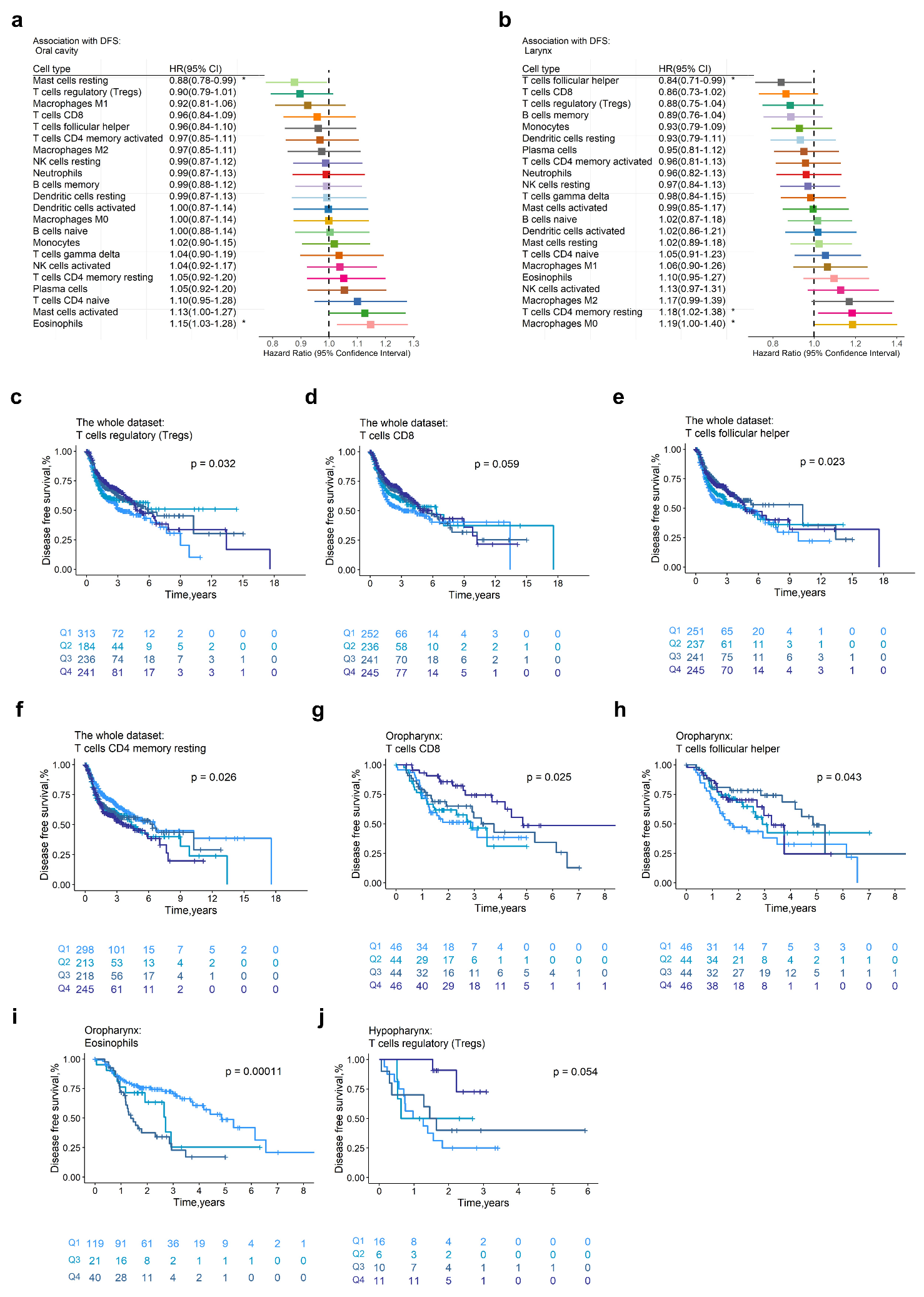


**Figure S4:** Association of immune cells with DFS across the whole dataset and four tumor subsites. Unadjusted HRs (boxes) and corresponding 95% confidence intervals (horizontal lines) for immune cell subtypes about the oral cavity subsite (a) and larynx subsite (b); Survival curve plots for the relative content of immune cells and DFS in the whole dataset (c-f), oropharynx subsite (g-i) and hypopharynx subsite (j). The immune cell subtypes are variables with *p* < 0.05 in the univariate Cox regression model. P-values are determined by the log-rank tests in Kaplan-Meier survival curves; DFS, disease-free survival.


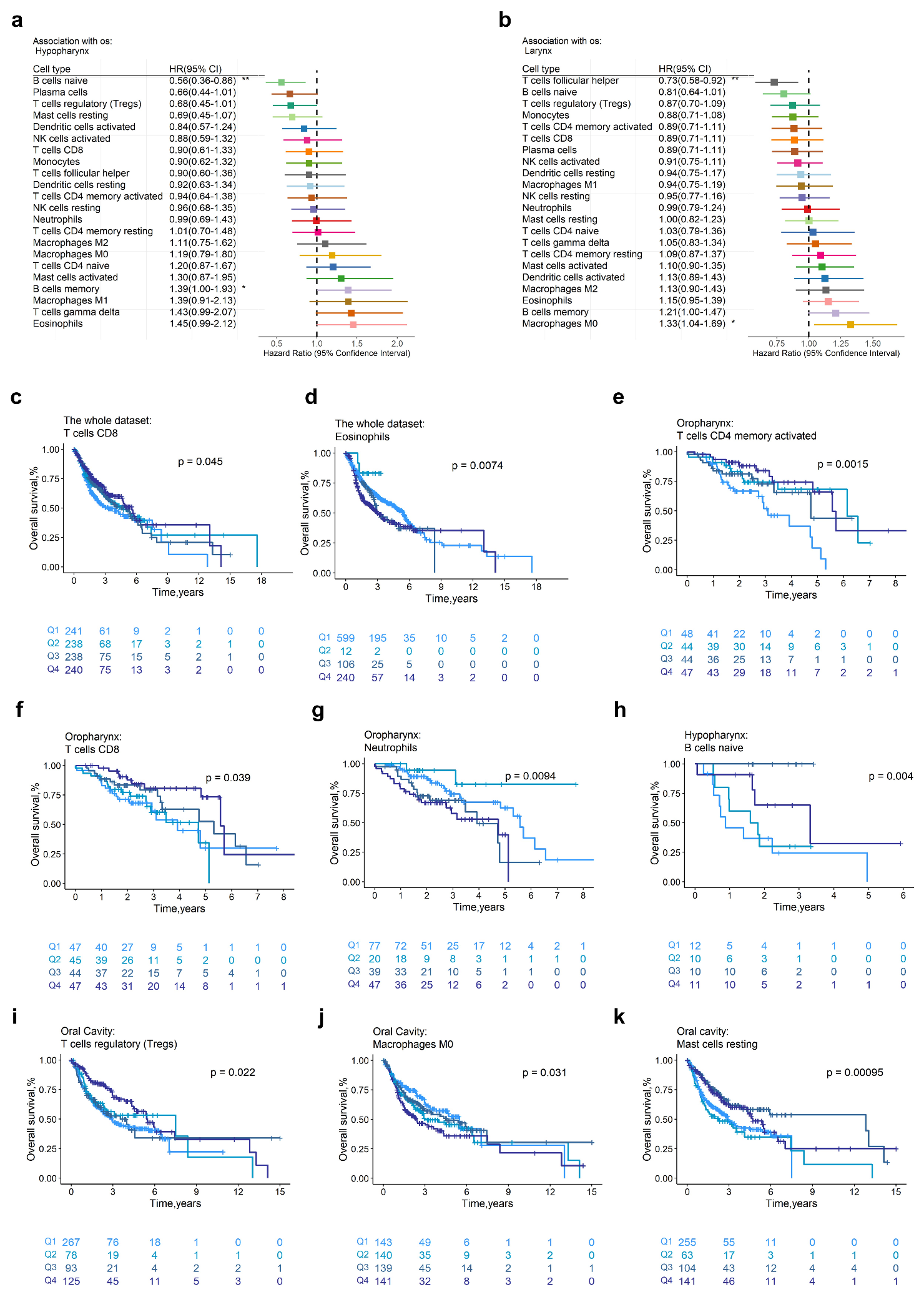


**Figure S5:** Association of immune cells with OS across the whole dataset and four tumor subsites. Unadjusted HRs (boxes) and corresponding 95% confidence intervals (horizontal lines) for immune cell subtypes about hypopharynx subsite (a) and larynx subsite (b); survival curve plots for the relative content of immune cells and OS in the whole dataset (c-d), oropharynx subsite (e-g), hypopharynx subsite (h), and oral cavity subsite (j-k). The immune cell subtypes are variables with *p*< 0.05 in the univariate Cox regression model. P-values are determined by the log-rank tests in Kaplan-Meier survival curves; OS, overall survival.


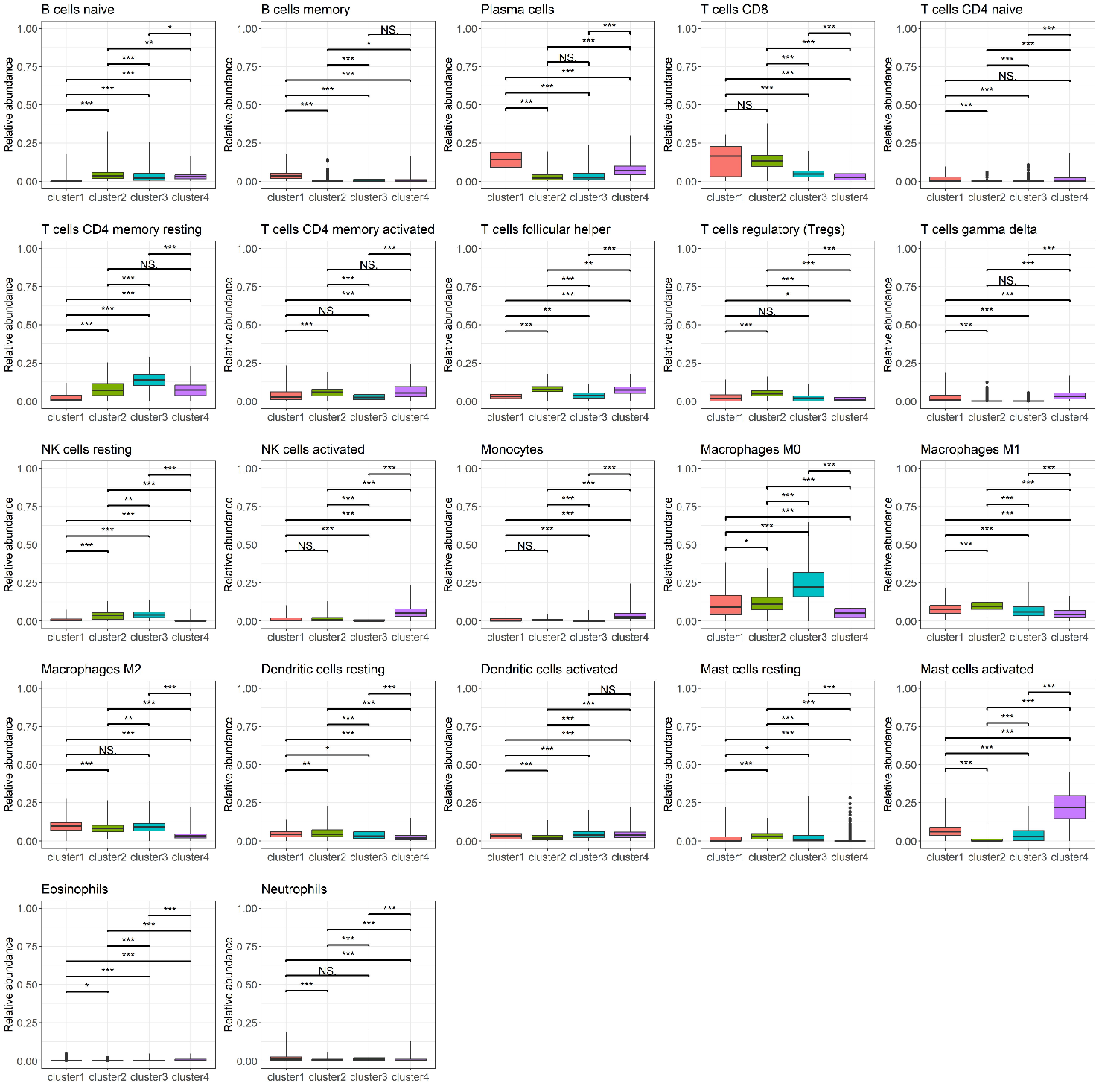


**Figure S6:** Box plots depicting the distribution of each immune cell subtype across four immune clusters. Wilcoxon test was used.


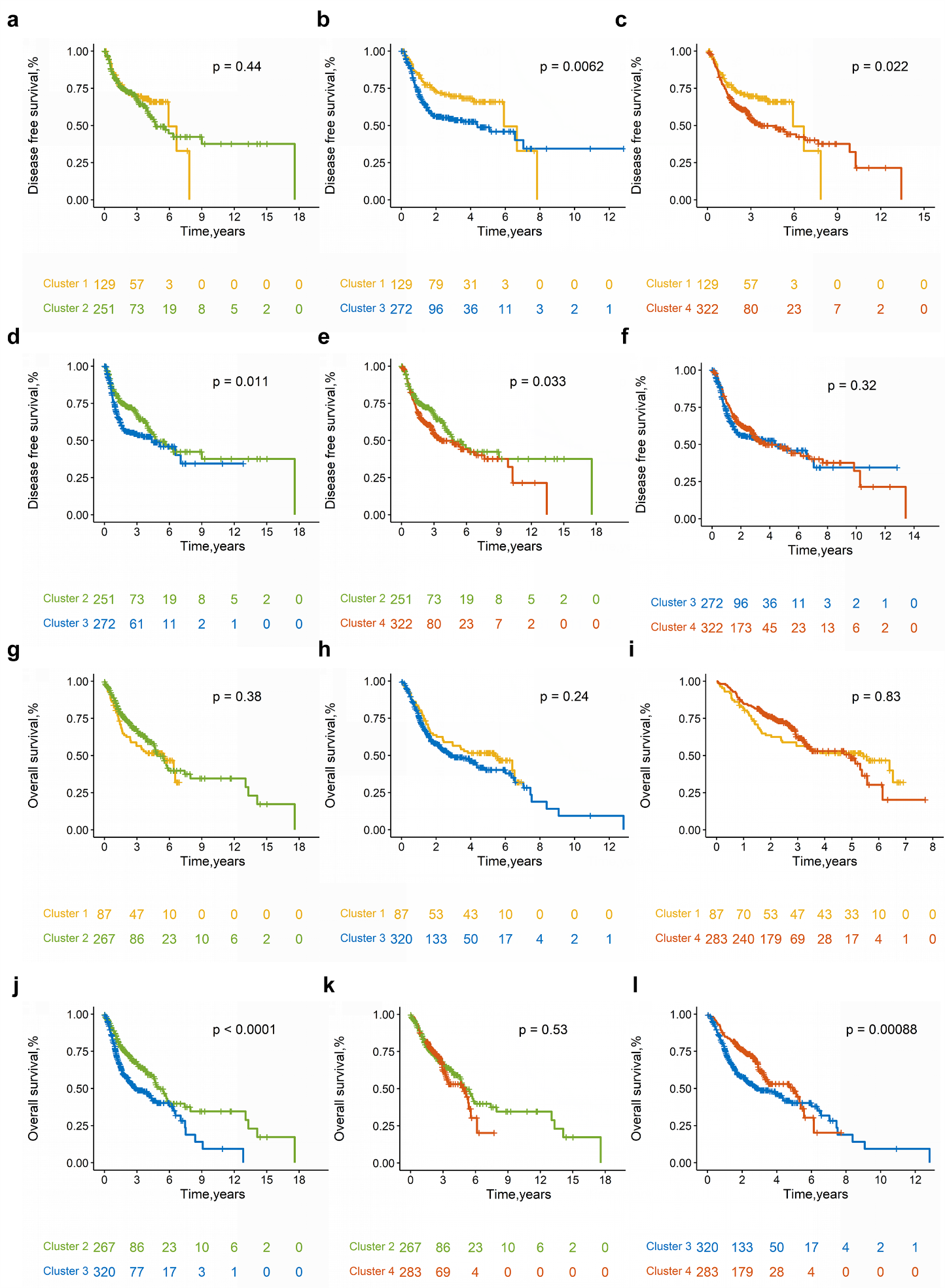


**Figure S7:** Survival analysis by pairwise comparison between four immune clusters; Disease-free survival (a-f); Overall survival (g-l). P-values are determined by the log-rank tests in Kaplan-Meier survival curves.


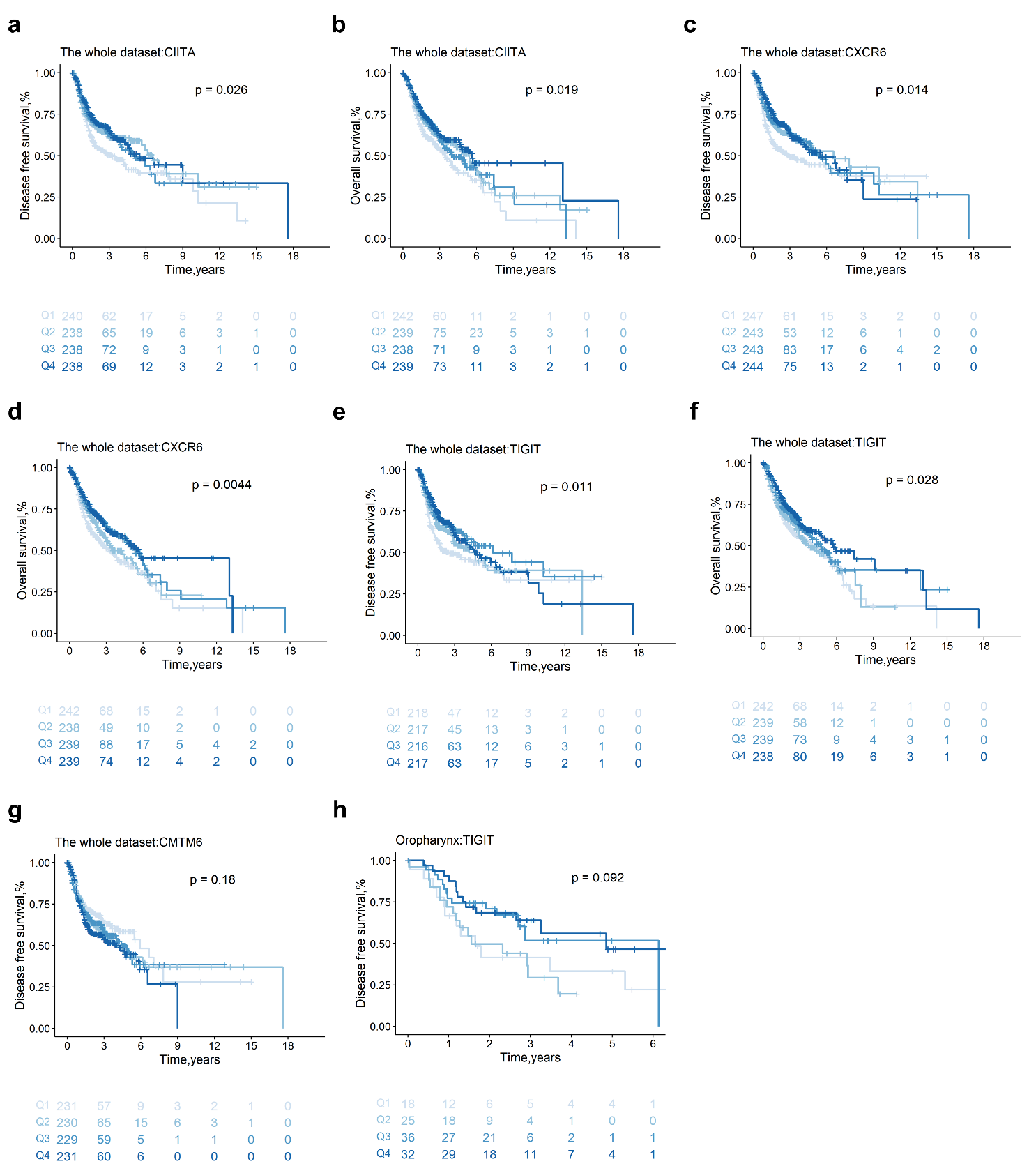


**Figure S8:** The association of immune gene expression with prognosis is illustrated by survival curves. Survival curve plots for CIITA (a-b), CXCR6 (c-d), and TIGIT (e-f) gene expressions with DFS and OS in the whole dataset, respectively; survival curve plots for CMTM6 gene expressions with DFS (g) in the whole dataset; survival curve plots for TIGIT gene expressions with DFS in the oropharynx subsite (h). The genes are variables with *p* < 0.05 in the univariate Cox regression model. P-values are determined by the log-rank tests in Kaplan-Meier survival curves; DFS, disease-free survival; OS, overall survival.


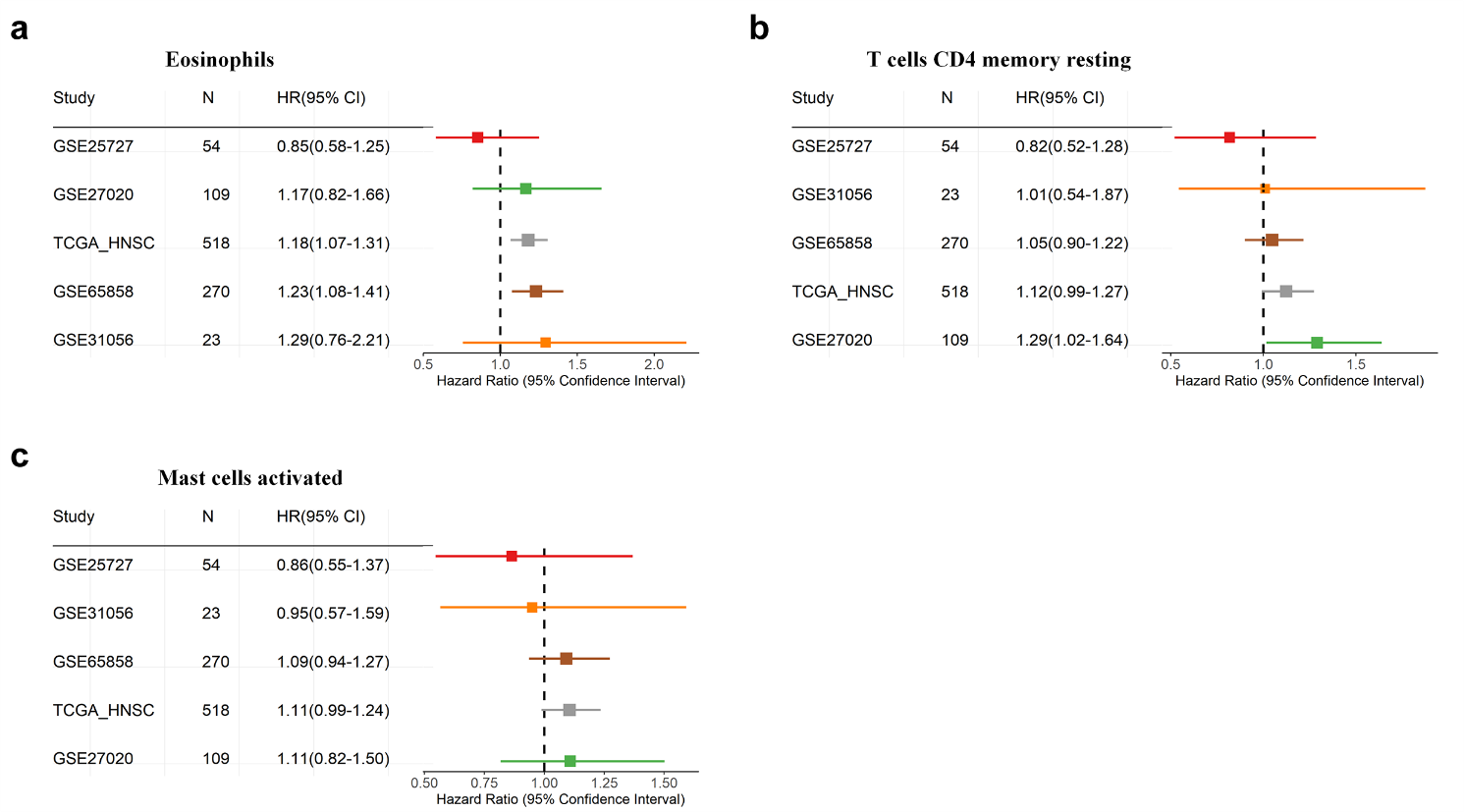


**Figure S9:** Forest plots show the meta-analysis results (disease-free survival as a clinical endpoint).


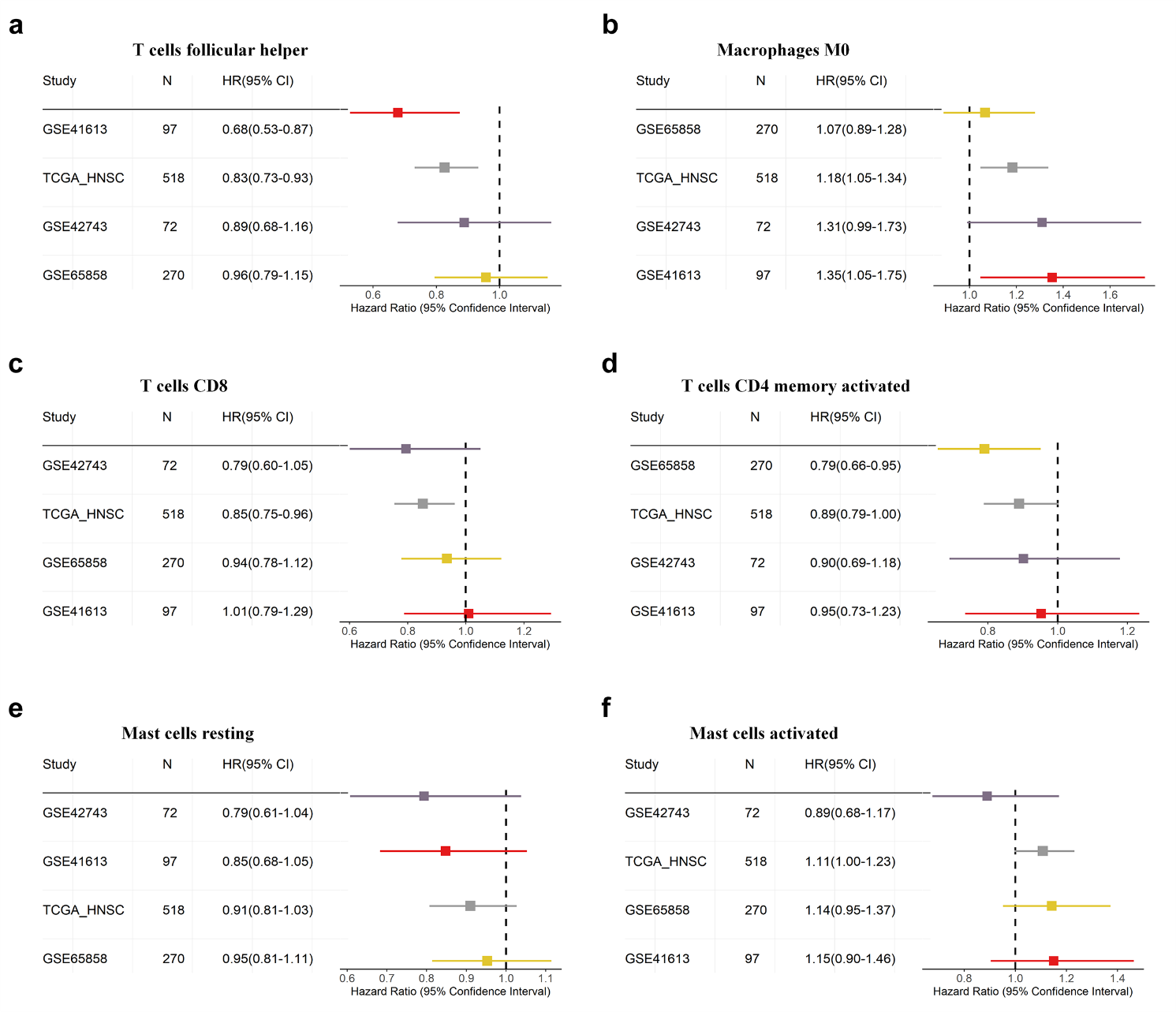


**Figure S10:** Forest plots show the meta-analysis results (overall survival as a clinical endpoint).


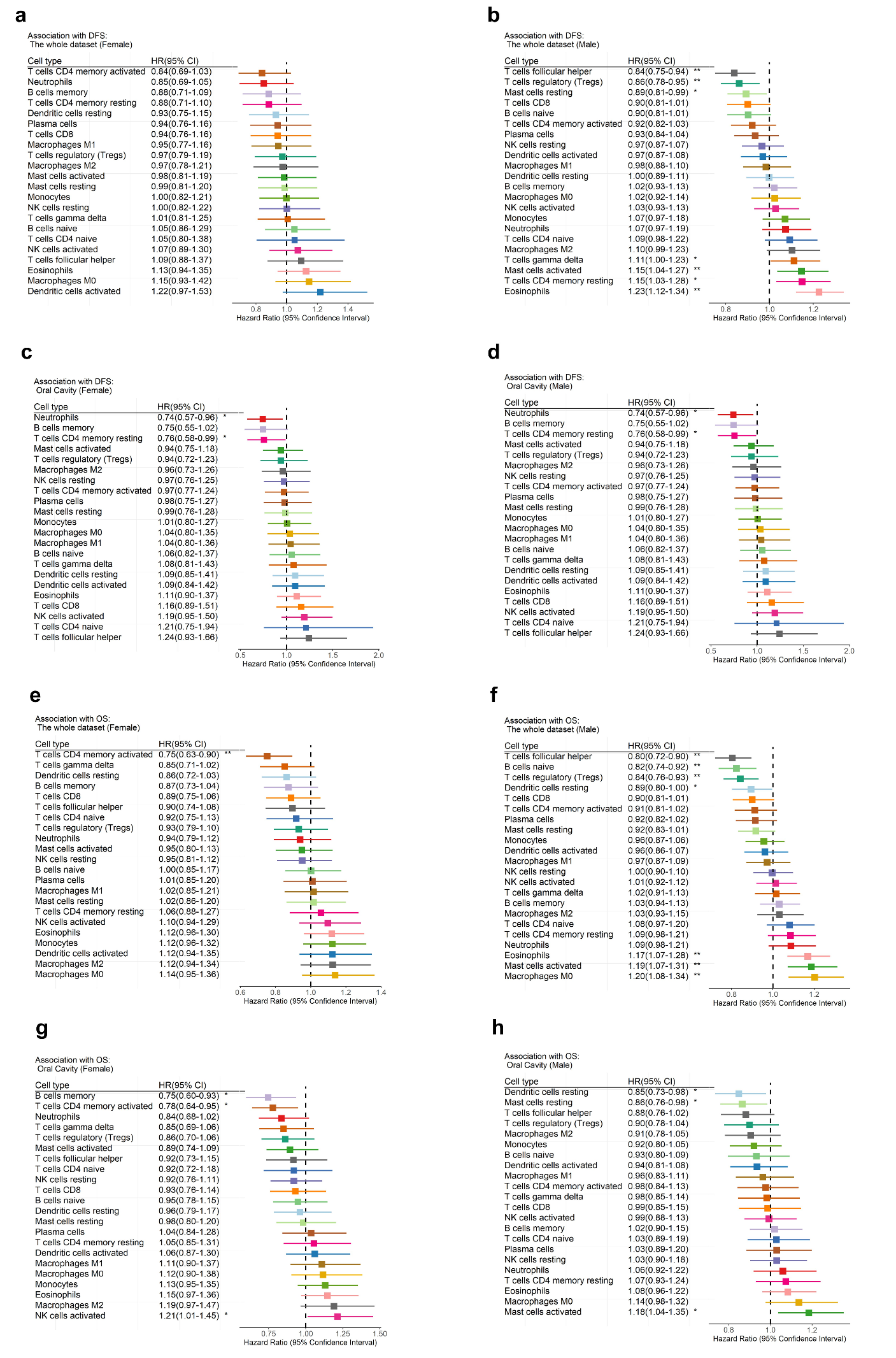


**Figure S11**: Forest plots of immune infiltration in men and women across the whole dataset and the oral cavity subsite (disease-free survival and overall survival as a clinical endpoint).
